# Phylogenomic Analyses Clarify True Species within the Butterfly Genus *Speyeria* despite Evidence of a Recent Adaptive Radiation

**DOI:** 10.3390/insects10070209

**Published:** 2019-07-17

**Authors:** Erin Thompson, Jason Baumsteiger, Ryan I. Hill

**Affiliations:** Department of Biological Sciences, University of the Pacific, Stockton, CA 95211, USA

**Keywords:** admixture, *Argynnis*, *Fabriciana*, principal component analysis, RADseq, *Viola*

## Abstract

When confronted with an adaptive radiation, considerable evidence is needed to resolve the evolutionary relationships of these closely related lineages. The North American genus *Speyeria* is one especially challenging radiation of butterflies due to potential signs of incomplete lineage sorting, ongoing hybridization, and similar morphological characters between species. Previous studies have found species to be paraphyletic and have been unable to disentangle taxa, often due to a lack of data and/or incomplete sampling. As a result, *Speyeria* remains unresolved. To achieve phylogenetic resolution of the genus, we conducted phylogenomic and population genomic analyses of all currently recognized North American *Speyeria* species, as well as several subspecies, using restriction-site-associated DNA sequencing (RADseq). Together, these analyses confirm the 16 canonical species, and clarify many internal relationships. However, a few relationships within *Speyeria* were poorly supported depending on the evolutionary model applied. This lack of resolution among certain taxa corroborates *Speyeria* is experiencing an ongoing adaptive radiation, with incomplete lineage sorting and lack of postzygotic reproductive barriers contributing to hybridization and further ambiguity. Given that many *Speyeria* taxa are under duress from anthropogenic factors, their legal protection must be viewed cautiously and on a case by case basis in order to properly conserve the diversity being generated.

## 1. Introduction

Understanding adaptive radiations helps clarify the evolutionary history and nature of different species, as well as the diversity of life [1,2,3]. Adaptive radiations represent the rapid diversification and formation of new species to fill newly available ecological niches. Many examples of adaptive radiations can be found across the globe. Excellent examples are the Hawaiian Silversword alliance (*Argyroxiphium, Dubautia* and *Wilkesia*) radiating into the available niches of Hawaii’s volcanic islands [4], and various cichlid fish radiating into different East African lakes, accumulating nearly two thousand unique species in the last ten million years [5]. Within insects, *Heliconius* butterflies are a clear example of an adaptive radiation driven by Müllerian mimicry and assortative mating based on color pattern [6,7,8]. Yet additional examples of adaptive radiations are needed to better understand the evolutionary factors which drive these unique speciation events.

Adaptive radiations give rise to relationships which are often difficult to resolve with phylogenetic methods [2]. There are several potential reasons for this. The first is the process of adaptive radiation which due to the rapid rate at which speciation occurs, leaves little genetic signal [9]. Second, incomplete lineage sorting (ILS), or the failure of all alleles to fully segregate into distinct lineages, can lead to gene trees that are discordant and not necessarily indicative of each new species [10,11,12,13]. Third, ongoing hybridization between new species can lead to reticulate patterns of evolution [14,15,16,17], violating the typical bifurcating assumption of phylogenetic methods. Any and/or all of these reasons can obscure the true evolutionary relationships.

One possible adaptive radiation in North America are butterflies from the genus *Speyeria*. *Speyeria* have a host-specific larval food plant relationship with *Viola* spp. [18], and are thought to have arrived in North America in the last 4–7 million years from Asia via the Bering land bridge. Upon arriving, they radiated onto the existing *Viola* diversity and ceased gene flow with Asian ancestors [19]. This genus is extremely diverse, comprising >100 taxa [20], with genitalia providing little help in identification and wing color pattern variation contributing to identification difficulties [21,22,23,24,25]. The color patterns of *Speyeria* species often co-vary geographically, especially among Western species, such that several species in a biogeographic area (i.e., mountain range or basin) resemble one another more than other taxonomically related subspecies. For example, in the California coast ranges near the Bay Area, *Speyeria callippe* and *S. coronis* (and also *S. zerene*) are brown to reddish brown on the ventral wing surface and “even many experienced observers are unable to determine whether some individuals are Callippe or Coronis Fritillaries” [26]. Yet in the Great Basin, *S. callippe* and *S coronis*, along with *S. zerene* and *S. egleis*, are much paler, with golden and greenish hues on the ventral wing surfaces blending with the vegetation there. Despite these challenges, available data indicate that *Speyeria* species show morphological, ecological and behavioral differences when in sympatry [27,28], and these traits should be associated with multi-locus differences indicative of species. Consistent with their rapid radiation, many taxa within the genus are interfertile and make viable hybrids in lab crosses, and examples of natural hybrids have been reported [29]. *Speyeria* are also of conservation concern, with many species in decline due to habitat loss and degradation from human activity [30,31]. In fact, several groups are federally listed endangered species such as *Speyeria callippe callippe*, *S. zerene behrensii*, and *S. zerene myrtleae* [32], while others are threatened, such as *S. idalia*, and *S. diana* [31], or imperiled such as *S. adiaste* [33,34].

The difficulties presented by the potential adaptive radiation discussed above, coupled with limited data and taxon sampling have led to unresolved phylogenies and uncertainty about the distinctiveness of species within *Speyeria*. Recent molecular phylogenetic investigations have helped confirm the monophyly of *Speyeria* and its relationship to close relatives in the genera *Argynnis* and *Fabriciana* [19,35,36], but relationships within *Speyeria* remain unresolved. McHugh et al. [23] included several taxa from the Western United States in an analysis focused on *S. zerene hippolyta*, and found most species studied to be paraphyletic (with the exception of *S. cybele*) suspecting that ILS gave rise to the patterns seen in their analysis. De Moya et al. [19] analyzed exemplars of all North American *Speyeria* species [20], and similar to McHugh et al. [23], found many species to be paraphyletic, with relationships between many species or species groups within *Speyeria* poorly supported. Campbell et al. [37] sampled *Speyeria* to validate methods using Next-Generation Sequencing (NGS) of non-model organisms, and to evaluate the relationships of *Speyeria* species. The five *Speyeria* species for which they sampled multiple exemplars had individuals cluster based on identification, but bootstrap values were <70, except for *S. hesperis*. Hill et al. [38] demonstrated that analyses using *CoI* recovered four *Speyeria* species at a smaller geographic scale compared with previous analyses [19,23]. Overall, analyses to date simply do not provide satisfactory validation of all North American *Speyeria* species or their inter-relationships due to a lack of comprehensive sampling and/or a finding of paraphyly among taxa.

Our goal here is thus to test the validity of current *Speyeria* species and to reconstruct the phylogenetic relationships of North American taxa. We did this by analyzing multiple exemplars of each of Pelham’s [20] 16 hypothesized *Speyeria* species, as well as *S. atlantis sorocko,* given it was treated as a full species by Hammond et al. [29]. We generated restriction-site-associated DNA sequence (RADseq) data from a single restriction enzyme and paired-end sequencing, yielding 2356 loci greater than 300 base pairs (bp) in length. These data were then used to investigate relationships and species validity of *Speyeria* with three independent analyses: phylogenomic, principal component analysis (PCA), and admixture analysis. 

## 2. Materials and Methods 

### 2.1. Sampling

Adult individuals from 17 hypothesized species were sampled across North America (Supplementary Table S1) using the taxonomy of Pelham [20]; except for *S. a. sorocko* [29]. Identifications were based on adult wing morphology [18,21,22,39,40,41,42,43,44]. Sampled adults were dissected on the day of capture, placed in 95–100% ethanol and finally into a −20–80 °C freezer. DNA was extracted from thorax or leg tissue using a QIAGEN DNeasy Blood and Tissue Kit (Germantown, MD, USA). DNA was quantified using a Qubit fluorometer and kit (Invitrogen, Carlsbad, CA, USA) and normalized to 5 ng/μL for library preparation. 

### 2.2. RADseq and de novo Assembly

DNA was digested with the restriction enzyme SbfI and paired-end 100 bp read libraries built using the protocol of Ali et al. [45]. All RAD sequencing data required a perfect barcode and partial restriction site match [45]. The genome assembler price [46] was used to create a de novo partial genome of RAD sequences from eight San Bruno Mountain *S. callippe* individuals. Sequences from all individuals in this study were then aligned to this reference assembly using the Burrows-Wheeler aligner program bwa (0.7.17-r1188) under default parameters. In samtools (1.7, htslib 1.7-2), view was used to create sorted and filtered files (-f 0 × 2), and rmdup was used to eliminate PCR duplicates to create the final Binary Alignment Map (BAM) files [47]. A minimum threshold of approximately 10× coverage (i.e., reads) was set per locus for all individuals included in our analyses (Supplementary Table S1). Information on the bioinformatic pipeline is covered in more detail in Baumsteiger et al. [48] and Ali et al. [45] and citations therein.

### 2.3. Phylogenomics

Phylogenomic analyses were conducted using four individuals per currently recognized North American *Speyeria* species [20], except *S. adiaste* with three individuals. In addition, two individuals were included to represent the putative species *S. atlantis sorocko* [29], for a total of 65 *Speyeria* individuals. In addition, eight outgroup individuals from Asia were included to test the position of *Speyeria clara* [19] and represent the closely related genera *Fabriciana* and *Argynnis* (Supplementary Table S1). 

Putative loci to be used in this study were generated from BAM files using angsd [49]. FASTA files for these loci were generated using default parameters (angsd -dofasta 2 -docounts 1). Because the total number of loci available exceeded the computational bounds of most phylogenomic programs, a subset of 50 loci was randomly chosen. All 50 loci were screened in seaview [50] and trimmed for missing sequence. The subprogram beauti within *beast [51] was used to set up the phylogenomic analysis parameters. This included a strict molecular clock, sampling 500 million generations, taking one sample every 10,000 generations and a Yule Model. Preliminary phylogenomic analyses explored different clock models (relaxed lognormal, relaxed exponential, random local, and strict) and found a strict clock to be the most appropriate based on effective sample size (ESS) values. Despite varying ESS values between analyses, overall topologies found between clock models were remarkably similar.

To explore the effects of increasing evolutionary substitution model parameterization on tree topology and ESS values, we performed three analyses: (1) Jukes Cantor (JC) for all 50 loci, (2) HKY for all 50 loci, or (3) parameterized by locus. Locus-specific substitution models were chosen based on the best corrected Akaike Information Criterion (AICc) value indicated by jmodeltest [52], and were independently entered into beauti (Supplementary Table S2). When a selected model was unavailable, the next best model indicated by jmodeltest was selected. Each of these three analyses was assessed in tracer [53] by reviewing ESS values, which are a measure of how well the Markov chains are mixing for each parameter. Species trees from all 50 loci were generated within *beast [51] and summarized with a tree annotator using a burn-in of 25% to give a final consensus species tree. Final visualization of each species tree was achieved with figtree [54]. 

### 2.4. Population Genomics

To further understand genetic variation among currently recognized species in North America and to validate these lineages using non-phylogenomic approaches, we used principal component and admixture analyses. Unlike the phylogenomic approach, these analyses make use of the entire suite of loci available from the de novo assembly. In these analyses, BAM files for each individual were used in angsd to identify polymorphic sites, infer major and minor alleles (doMajorMinor 1), estimate allele frequencies (doMaf 2), and retain single nucleotide polymorphisms (SNPs) with a minor allele frequency of at least 0.05 (minMaf). PCAs were generated by creating covariance matrices from genotypes called by angsd using the NGSCOVAR function within ngstools and plotting results in R [55,56]. Admixture analyses were completed by generating BEAGLE input files in angsd (with the same parameters as the PCA) and running those input files in ngsadmix. Analyses were conducted from K = 2 to K = 20 with minimum 10 iterations. The optimal number of clusters, K, was assessed using delta-K, following the method of Evanno et al. [57]. Results were processed and visualized using the pophelper [58] package (2.2.9) in R 3.5.2 (Vienna, Austria).

## 3. Results

### 3.1. RADseq

The de novo assembly generated using eight *S. callippe* individuals returned a partial genome of 2356 loci, with a minimum of 300 bp in length and average of 570 bp. All individuals were aligned to this partial genome, with no ingroup individual containing fewer than 34.5 k reads, and with 54 of 65 individuals having greater than 100 k reads (mean = 186 k). All sequence data used in our analyses are available at National Center for Biotechnology Information (NCBI) with the following BioProject accession number PRJNA552154. Final BAM files for all individuals from North America revealed 51,245 SNPs were available for population genomic analyses. The final set of 50 loci used for phylogenomic analyses had an average sequence length of 345 bp and average minimum of 199 bp for all loci across all 73 individuals (Supplementary Table S2). This final phylogenomic dataset was comprised of 17,933 total sites, with 905 being parsimony informative (5.0%).

### 3.2. Phylogenomics

The analysis using a relatively simple evolutionary model, JC, on all 50 loci showed high posterior probabilities (>0.95) for 15 of 17 hypothesized species (Supplementary Figure S1). *Speyeria egleis* had a low posterior probability of 0.56. The ESS values for the posterior, species coalescent and Yule Model were all >200 which exceeds the *BEAST conservative recommendation of having ESS >100 [51]; however, tree likelihood ESS = 68. Further details (see Supplementary Figure S1) are included below when describing the HKY and parameterized analyses.

**Figure 1:**
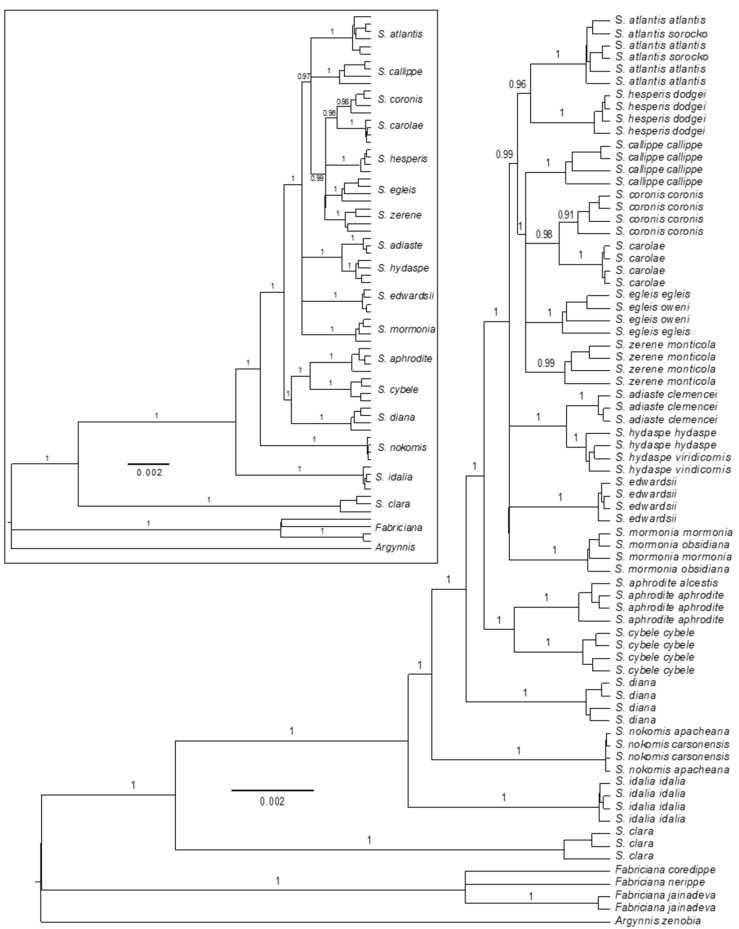
Phylogenomic results for analyses with parameter rich locus-specific substitution models and HKY (inset) for all 50 loci. Posterior values are omitted within species and branches are collapsed for interspecies nodes with <0.95, except for *S. coronis* in the parameterized tree.

The tree constructed with the HKY model of evolution recovered 16 of 17 species with high posterior probabilities (>0.95; Figure 1 inset). The results were very similar to JC but here, *Speyeria egleis* was monophyletic (poster probability = 1.0) and the position of *S. diana* differed. The ESS values for the posterior, species coalescent, and Yule Model were all greater than 200, with tree likelihood = 91. *Speyeria* was clearly monophyletic, with *S. clara* sister to all remaining *Speyeria*. *Fabriciana* was monophyletic but its position was unresolved relative to *Speyeria* and *Argynnis*. Two species (*S. idalia* and *S. nokomis*) branched basally to the remainder of the tree. There were two main lineages after *S. idalia* and *S. nokomis*: one consisted of *S. diana*, *S. aphrodite* and *S. cybele* and the other represented the “callippe-group” of Hammond et al. [29] which includes: *S. mormonia*, *S. adiaste*, *S. hydaspe*, *S. edwardsii*, *S. hesperis*, *S. atlantis*, *S. egleis*, *S. zerene*, *S. carolae*, *S. coronis*, and *S. callippe*. The HKY analysis resulted in *S. diana* being sister to the pair *S. aphrodite* + *S. cybele* whereas JC had *S. diana* as sister to the remaining species. This change was not completely unexpected given the poor support for short branches in the JC analysis. Within the callippe-*group*, well-resolved sister species were *S. adiaste* + *S. hydaspe*, and *S. coronis* + *S. carolae*. Compared with JC, other internal relationships in the callippe-group involving *S. atlantis*, *S. hesperis*, *S. callippe*, *S. carolae*, *S. coronis*, *S. egleis* and *S. zerene* showed improved resolution. Finally, individuals of *S. atlantis sorocko* were paraphyletic with *S. atlantis*, but the branch leading to *S. atlantis* and *S. a. sorocko* had a posterior probability of 1.0.

In the final tree constructed using locus-specific substitution models, 15 of 17 hypothesized species were found to be monophyletic with high posterior probabilities (>0.95, Figure 1) and there was increased resolution of inter-relationships compared to the other analyses. The ESS values for the posterior, species coalescent, and Yule Model were all >200 and the tree likelihood >100. The posterior probability for monophyly of *S. coronis* was 0.91. Relationships for *S. clara*, *Fabriciana*, *Argynnis*, and *S. idalia* and *S. nokomis* were the same as in the other two analyses. The position of *S. diana* was strongly supported as sister to the remaining *Speyeria*, with *S. cybele* and *S. aphrodite* recovered as sister to the callippe-group. Within the callippe-group, a lineage consisting of *S. callippe*, *S. coronis*, *S. carolae*, *S. egleis*, and *S. zerene* was strongly supported (posterior probability = 1.0). In addition, an important change was the sister species relationship of *S. atlantis* + *S. hesperis* not seen in the other analyses. The samples of *S. atlantis sorocko* were again paraphyletic with *S. atlantis*, and *S. atlantis* plus *S. a. sorocko* had a posterior probability of 1.0. *Speyeria coronis* and *S. carolae* were recovered as sister taxa, as were *S. adiaste* and *S. hydaspe*.

### 3.3. Population Genomics: PCA

**Figure 2:**
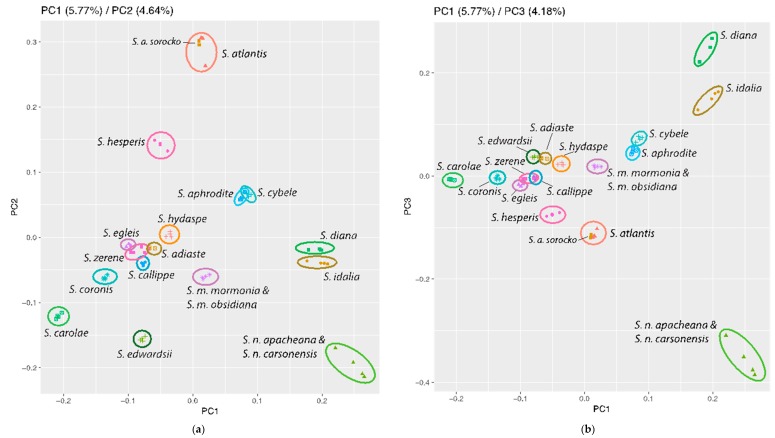
Principal component analyses of 17 hypothesized *Speyeria* species: (**a**) PC 1 vs. PC 2 which account for 5.8% and 4.6% of the variation in the overall dataset respectively; (**b**) PC 1 vs. PC 3 (4.2%).

The principal components analysis using the entire data set of 2356 loci identified 16 of 17 contemporary lineages as non-overlapping points when plotting the first three principal components (PCs, Figure 2). Of the total variation, PC1 accounted for 5.8%, whereas PC2 accounted for 4.6% and PC3, 4.2%. The relatively gradual decrease in the amount of variation explained by the PCs (Supplementary Figure S2), indicates the total variation in the dataset was not encapsulated within the first few PCs. Despite this, individuals from each morphological species cluster with one another, largely supporting their species status. For example, *S. edwardsii* and *S. carolae* are well-separated from other taxa with PC1 and PC2. *Speyeria idalia, S. nokomis* and *S. diana* are separated from the remainder of the genus on PC1 and PC3 (Figure 2). *Speyeria cybele* and *S. aphrodite* are very close or overlap on PC1 and PC2, but do not overlap on PC3. There was obvious overlap between *S. a. sorocko* and *S. a. atlantis*, whereas *S. hesperis* were well-separated from *S. atlantis*. Several species in the callippe-group were closer together, with some species overlapping (e.g., *S. zerene* and *S. callippe*), but these species become more distinguishable with further PC sampling.

### 3.4. Population Genomics: Admixture

**Figure 3:**
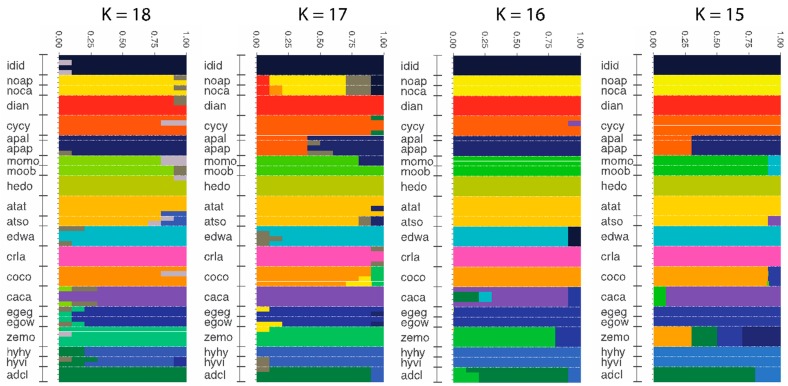
Admixture analysis results for Ks which recover the reproductively isolated species *S. idalia* and *S. diana* as distinct clusters. The four-letter codes indicting *Speyeria* species and subspecies are: idid = *idalia idalia*, noap = *nokomis apacheana*, noca = *nokomis carsonensis*, dian = *diana*, cycy = *cybele cybele*, apal = *aphrodite alcestis*, apap = *aphrodite aphrodite*, momo = *mormonia mormonia*, moob = *mormonia obsidiana*, hedo = *hesperis dodgei*, atat = *atlantis atlantis*, atso = *atlantis sorocko*, edwa = *edwardsii*, crla = *carolae*, coco = *coronis coronis*, caca = *callippe callippe*, egeg = *egleis egleis*, egow = *egleis oweni*, zemo = *zerene monticola*, hyhy = *hydaspe hydaspe*, hyvi = *hydaspe viridicornis*, adcl = *adiaste clemencei*.

A log-likelihood cluster evaluation indicated the highest delta-K for K = 2, followed by K = 4 and K = 6 (Supplementary Figure S3). However, delta-K values were low (<4). Clustering for delta K = 2, 4 and 6 roughly match higher-level patterns seen in the phylogenomic analyses (Supplementary Figure S4), with basally branching taxa separated from more recent splitting groups. However, these Ks do not recover the morphologically distinct and reproductively isolated taxa *S. idalia*, *S. diana*, and *S. nokomis.* The number of clusters with the next best delta-K values were K = 16–18 (Figure 3). Here, *S. idalia* and *S. diana* are both distinct clusters in all three Ks (*S. nokomis* in K = 16 and 18). This is consistent with the result of Hammond et al. [29], which found these species do not make fertile offspring when crossed with other *Speyeria*. The higher values of K (K = 16–18) also showed good resolution of the remaining *Speyeria* species. Therefore, we followed Pritchard et al. [59] and evaluated clusters consistent with previous knowledge of the genus. 

At K = 16, clusters coincided with sensible biological patterns and contemporary species designations within *Speyeria*, while maintaining reproductively isolated taxa as distinct clusters. For example, 14 of the 17 putative species can be clearly defined as unique using a threshold of 75% assignment to a particular cluster, including *S. idalia, S. nokomis and S. diana*. *Speyeria callippe* and *S. adiaste* are also well-differentiated at close to the 75% threshold. When K = 15 is selected, again 14 of 17 contemporary lineages formed distinct clusters but *S. aphrodite* and *S. zerene* were not recovered. To account for the possibility of cryptic species within the genus, we examined the results at K = 17–18. At K = 17, *S. nokomis* and *S. aphrodite* have increased levels of admixture rather than *S. atlantis sorocko* or one of the other pairs of subspecies being a distinct cluster. The analysis at K = 17, with 13 of 17 lineages resolved, represents the start of decreasing resolution for this and subsequent Ks. For example, at K = 18, the 17th and 18th clusters are scattered across many species, rather than being correlated with any known subspecies or species, largely keeping intact the distinctiveness of the 16 hypothesized species identified at K = 16. In sum, this approach, using biological intuition based on reproductive isolation, results in K = 16 being the best fit to the data.

## 4. Discussion

Our independent phylogenomic, admixture, and PCA analyses, when taken together, indicate there are multi-locus differences among the 16 *Speyeria* species in Pelham’s [20] catalog, and help to disentangle the paraphyly indicated in previous studies [19,23,29]. Our analyses corroborate relationships found by others with similar data [37] and combined with previous phylogenetic work [19,37], illustrate that these taxa are genetically distinct with genome-wide markers, despite evidence of a lack of postzygotic barriers amongst many species [29]. This information is invaluable for conservation efforts going forward as the clear diagnosis of these species can now be used to justify protection under the Endangered Species Act. Additionally, with properly defined species, identification of putative subspecies and distinct population segments can be better authenticated in future studies, thereby increasing both our knowledge and protection of the growing diversity within this genus of butterflies.

Overall, our results clarify species designations of *Speyeria* which have been found to be paraphyletic or had their distinctiveness debated on morphological grounds. One example is *S. carolae* and *S. coronis*, which our three independent analyses found to be distinct and closely related species, corroborating previous biosystematic work [39] and the current taxonomic arrangement [20]. A second example is *S. atlantis* and *S. hesperis.* Multiple sources regard these as distinct species [18,20,40,41,44,60,61], whereas others consider *S. hesperis* as a junior synonym of *S. atlantis* [26,29,42]. Our combined analyses found these taxa to be distinct, with some phylogenomic results not even placing them as sister taxa (Figure 1). Based on our results, *S. atlantis* and *S. hesperis* should continue to be recognized as separate species [20,44]. Finally, *Speyeria atlantis sorocko* was never recovered as a genetic cluster or monophyletic entity in any analysis and should, therefore, be maintained as a subspecies of *S. atlantis*, rather than given species status as in Hammond et. al. [29]. Further work is still needed on *S. atlantis* and *S. hesperis* to investigate whether *S. a. hollandi*, which was recognized as a species by Hammond et al. [29], warrants species status.

The phylogenomic results of this study also shed light on the hypothesized callippe- and cybele-groups of Hammond et al. [29], corroborating the callippe-group but not the cybele-group. Our analyses show the cybele-group is paraphyletic and place *S. aphrodite* + *S. cybele* as a lineage sister to members of Hammond’s callippe-group [29], similar to de Moya et al. [19] (Figure 1). These results indicate that the genitalic characters cited as defining the callippe-group [29] are good synapomorphies, but those for the cybele-group are symplesiomorphies.

Although our analyses revealed strong patterns in the data, there were some species that failed to be recovered depending on the analysis type, or specific PCs or Ks. Species from the callippe-group, and especially, the most recent lineage within it, were most often the species not recovered, which reflects their recent origin. For example, in the phylogenomic analyses, *S. coronis* and *S. egleis* both had low support, each in a different analysis (parameterized for *S. coronis*, Figure 1; JC for *S. egleis*, Supplementary Figure S1). In the PCA, the greatest overlap involved the callippe-group taxa and the sister species *S. cybele* and *S. aphrodite* (Figure 2). In the admixture analysis for K = 15, *S. aphrodite*, and especially *S. zerene*, showed significant levels of admixture with *S. cybele* and callippe-group species, respectively. At K = 16, *S. aphrodite and S. zerene* appeared distinct, although *S. callippe* and *S. adiaste* showed more admixture than at K = 15. However, these patterns were not consistent across all Ks, with only one K reflecting an anomaly.

Whereas the effects of evolutionary modeling on phylogenetic resolution can be seen in our phylogenomic analyses, they are minimal in changing the phylogeny of *Speyeria*, given the consistent topological patterns. Our results clearly indicate the parameterized tree as the best topological representation of the *Speyeria* phylogeny because it had the best ESS, and the highest posterior probabilities (Figure 1), despite the low posterior for *S. coronis*. In all analyses, *S. idalia* and *S. nokomis* were the first and second lineages, respectively, to split from the rest of *Speyeria*. However, the position of *S. diana* varied from the third lineage to branch off of North American *Speyeria*, to being sister to *S. cybele* + *S. aphrodite. Speyeria cybele* and *S. aphrodite* were consistently sister taxa, as were *S. coronis* and *S. carolae* and *S. adiaste* and *S. hydaspe.* The callippe-group was consistently monophyletic, with its internal relationships increasingly resolved with increased parameterization. Thus, the results presented here are largely congruent with, and have helped clarify, previous work that contained low support values [19,37]. Further work analyzing different model assumptions may be able to improve topological resolution. However, given that our genomic analyses support species designations, the short branch lengths and low posterior values for interior branches strongly corroborate *Speyeria* as a recent, rapid radiation [19].

Similar to our phylogenomic analyses, the PC and admixture results are not definitive on their own but tell a compelling story collectively and use a larger proportion of the available genomic data. For example, each PC in our analysis does not show large amounts of variation seen among species in other studies [for example see 48] but the 16 hypothesized species are clearly evident. The admixture results are no different, with support for each K being relatively low but again, the 16 currently recognized species are definable at K = 16. Additionally, the PC analysis showed patterns present in the phylogenomic analysis, with early branching species more widely separated, reflecting they are well-differentiated and strongly reproductively isolated [19,29,37,62]. Furthermore, well-supported sister species were some of the taxa showing the most overlap (i.e., *S. cybele* and *S. aphrodite*) in the PC analyses. Species in the callippe-group lineage identified in the parameterized tree comprising *S. callippe*, *S. coronis*, *S. carolae*, *S. egleis* and *S. zerene* (Figure 1) showed the greatest overlap. We consider the overlap between species and the small amount of variation explained by the first three PCs, to again be signatures of recent adaptive radiation, in that these ecologically and morphologically distinctive species are not vastly different from one another genetically [19,20,23,29,37,63].

The reduced support of patterns in the admixture and PC analyses are consistent with a genus undergoing an adaptive radiation. Thus, hybridization is probable given the relatively short time frame in which speciation is occurring and has been found in the callippe-group. Given that the callippe-group taxa, and *S. aphrodite* and *S. cybele* have been shown to produce viable offspring and few Haldane effects when crossed with each other [29], the admixture and PCA patterns could be interpreted as indicating introgression from hybridization. However, incomplete lineage sorting could also produce similar patterns. We can only conclude that these species underwent rapid radiation and are still in the process of evolving genetic incompatabilities.

The kind of analyses applied here, coupled with the paraphyly found in *Speyeria* in other analyses, and the disagreement on designations for the various taxa, have important implications for conservation work and our understanding of *Speyeria* evolution. Since the approaches undertaken here reaffirmed species of *Speyeria* based on a comprehensive biological approach and improved phylogenetic resolution, they can be expanded to test hypotheses of evolution in the genus. Given our sampling of the wide geographic and morphological variation within *Speyeria* is incomplete, we see our results as a solid taxonomic foundation. Future studies can focus on the extensive subspecies structure currently proposed in many of the canonical species, evaluating whether any subspecies warrants a change in status. Additionally, the population genomics methods employed here can be used to evaluate a number of imperiled populations, such as the endangered *S. zerene hippolyta* or *S. callippe callippe* [38], which can help in the conservation of the increasing diversity within *Speyeria*. Finally, with recognition of this adaptive radiation, biologists can monitor and better understand the fundamental evolutionary tenets behind how an adaptive radiation occurs and how diversification leads to speciation.

## 5. Conclusions

Overall, our results validated the 16 canonical *Speyeria* species, and indicated that *S. a. sorocko* is not well-differentiated from other *S. atlantis*. North American *Speyeria* were clearly monophyletic with consistent topological patterns for several relationships, including the position of *S. idalia* and *S. nokomis*, the monophyly of the callippe-group, and sister relationships for *S. cybele* and *S. aphrodite*, *S. adiaste* and *S. hydaspe*, and *S. coronis* and *S. carolae*. However, there was strong evidence in the weakly supported relationships, and admixture and PCA overlap among the more recently diverged taxa, indicating that *Speyeria* are undergoing a recent and apparently ongoing adaptive radiation within North America. Special attention must, therefore, be paid to these emerging species and the conditions leading to this diversification, so that we might conserve the diversity being generated both now and in the future.

## Supplementary Materials

The following are available online at https://www.mdpi.com/2075-4450/10/7/209/s1, Figure S1: Jukes Cantor tree, Figure S2: Scree plot, Figure S3: Plot of delta-Ks, Figure S4: Admixture plots, Table S1: Sample list and reads per locus, Table S2: Information on loci analyzed.
