# Supplementary material for: Phylogenomic Analyses Clarify True Species within the Butterfly Genus Speyeria despite Evidence of a Recent Adaptive Radiation
Source: Insects. 2019 Jul 17;10(7):209. doi: 10.3390/insects10070209 (PMC6681192; doi:10.3390/insects10070209)
Supplement: Supplementary file 1 [file insects-10-00209-s001.zip › insects533876-suppl/Figure S1-S4.docx]

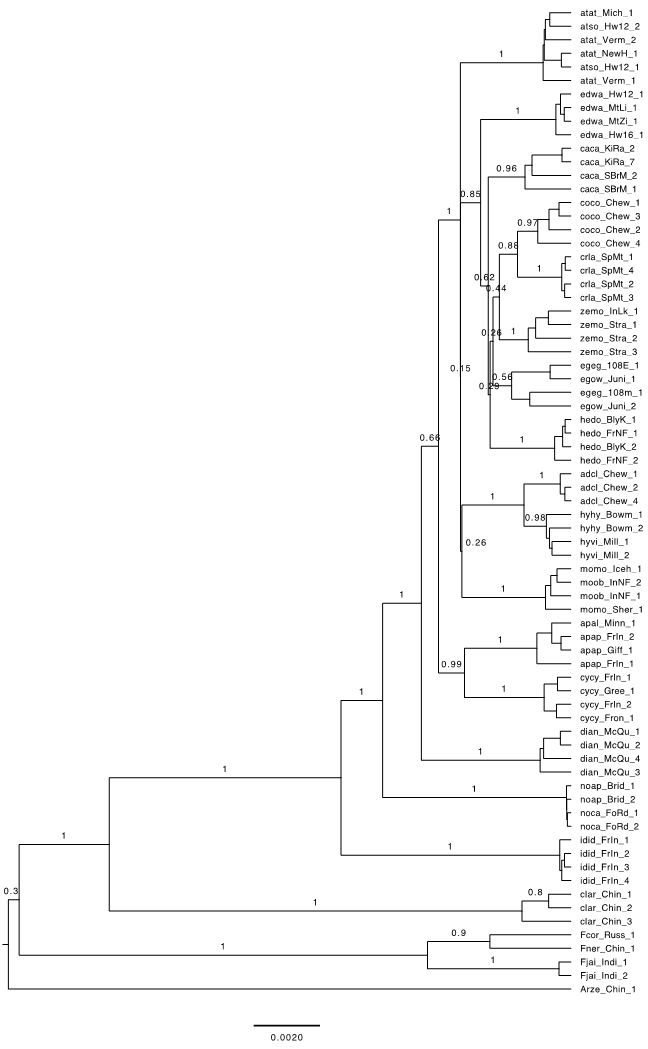


**Figure S1.** Phylogenetic tree created with Jukes Cantor model for all 50 loci.

**Figure S2.** Scree plot showing decrease in variance explained by principal components after Eigen decomposition of covariate data.

**Figure S3.** Plot of delta-K’s indicated by the Evanno method.

**
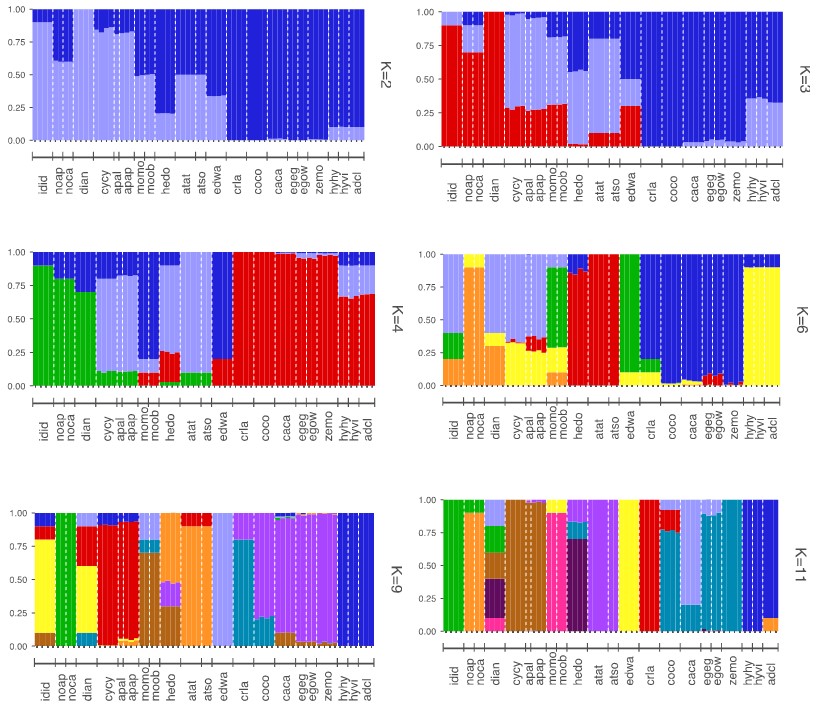
**

**Figure S4.** Admixture plots with higher delta-K indicated by Evanno method. Although these values of K have relatively high delta-K, the delta-K values are all low (<4). These K’s correlate with higher level structure in phylogenetic analyses, or do not strongly recover reproductively isolated species such as *S. idalia* and *S. diana*.
