# Supplementary material for: Phylogenomic Analyses Clarify True Species within the Butterfly Genus Speyeria despite Evidence of a Recent Adaptive Radiation
Source: Insects. 2019 Jul 17;10(7):209. doi: 10.3390/insects10070209 (PMC6681192; doi:10.3390/insects10070209)
Supplement: Supplementary file 1 [file insects-10-00209-s001.zip › insects533876-suppl/Table S1.docx]

**Table S1.** List of *Speyeria* and outgroup individuals sampled, and the average number of reads per locus for loci used in the phylogenomic analyses.

| **Individual ID** | **Voucher Number** | **Species** | **Subspecies** | **Country, State/Prov, County** | **Avg. Read/Locus** |
| --- | --- | --- | --- | --- | --- |
| adcl_Chew_1 | RIH2329 | *adiaste* | *clemencei* | USA, CA, Monterey Co. | 149.1 |
| adcl_Chew_2 | SCC272 | *adiaste* | *clemencei* | USA, CA, Mendocino Co. | 35.3 |
| adcl_Chew_4 | RIH2557 | *adiaste* | *clemencei* | USA, CA, Mendocino Co. | 66.3 |
| apal_Minn_1 | CRT041 | *aphrodite* | *alcestis* | USA, MN, St. Louis Co. | 54.5 |
| apap_FrIn_1 | WS13-18-b | *aphrodite* | *aphrodite* | USA, PA, Lebanon Co. | 30.9 |
| apap_FrIn_2 | WS13-19-b | *aphrodite* | *aphrodite* | USA, PA, Lebanon Co. | 49.0 |
| apap_Giff_1 | WS13-14 | *aphrodite* | *aphrodite* | USA, PA, York Co. | 19.6 |
| atat_NewH_1 | RIH2602 | *atlantis* | *atlantis* | USA, White Mtns., NH. | 48.5 |
| atat_Verm_1 | VT001 | *atlantis* | *atlantis* | USA, VT, Addison Co. | 65.6 |
| atat_Verm_2 | VT002-b | *atlantis* | *atlantis* | USA, VT, Addison Co. | 39.5 |
| atat_Mich_1 | CRT035 | *atlantis* | *atlantis* | USA, MI, Schoolcraft Co. | 69.4 |
| atso_Hw12_1 | RIH2744 | *atlantis* | *sorocko* | USA, CO, Gunnison Co. | 69.4 |
| atso_Hw12_2 | RIH2750 | *atlantis* | *sorocko* | USA, CO, Gunnison Co. | 64.6 |
| caca_KiRa_2 | SCC-49 | *callippe* | *callippe* | USA, CA, Solano Co. | 105.9 |
| caca_KiRa_7 | SCC-47B | *callippe* | *callippe* | USA, CA, Solano Co. | 108.1 |
| caca_SBrM_1 | SCC-21 | *callippe* | *callippe* | USA, CA, San Mateo Co. | 75.2 |
| caca_SBrM_2 | SCC-19 | *callippe* | *callippe* | USA, CA, San Mateo Co. | 72.8 |
| crla_SpMt_1 | CRT090 | *carolae* |  | USA, NV, Clark Co. | 72.6 |
| crla_SpMt_2 | CRT092 | *carolae* |  | USA, NV, Clark Co. | 133.6 |
| crla_SpMt_3 | CRT089 | *carolae* |  | USA, NV, Clark Co. | 130.8 |
| crla_SpMt_4 | CRT091 | *carolae* |  | USA, NV, Clark Co. | 116.7 |
| coco_Chew_1 | RIH2333 | *coronis* | *coronis* | USA, CA, Monterey Co. | 90.8 |
| coco_Chew_2 | SCC-276 | *coronis* | *coronis* | USA, CA, Monterey Co. | 180.8 |
| coco_Chew_3 | SCC-277 | *coronis* | *coronis* | USA, CA, Monterey Co. | 175.9 |
| coco_Chew_4 | SCC-279 | *coronis* | *coronis* | USA, CA, Monterey Co. | 183.0 |
| cycy_Fron_1 | WKS-S-02 | *cybele* | *cybele* | USA, VA, Warren Co. | 98.5 |
| cycy_Gree_1 | WMNF-01 | *cybele* | *cybele* | USA, NH, Carrol Co. | 38.1 |
| cycy_FrIn_1 | WS13-15 | *cybele* | *cybele* | USA, PA, Lebanon Co. | 63.5 |
| cycy_FrIn_2 | WS13-17 | *cybele* | *cybele* | USA, PA, Lebanon Co. | 109.0 |
| dian_McQu_1 | WS13-33 | *diana* |  | USA, TN, Johnson Co. | 73.0 |
| dian_McQu_2 | WS13-26 | *diana* |  | USA, TN, Johnson Co. | 75.8 |
| dian_McQu_3 | WS13-31 | *diana* |  | USA, TN, Johnson Co. | 64.5 |
| dian_McQu_4 | WS13-32 | *diana* |  | USA, TN, Johnson Co. | 26.6 |
| edwa_Hw12_1 | RIH2767 | *edwardsii* |  | USA, CO, Gunnison Co. | 72.3 |
| edwa_Hw16_1 | RIH2819 | *edwardsii* |  | USA, WY, Johnson Co. | 88.4 |
| edwa_MtZi_1 | RIH2818 | *edwardsii* |  | USA, CO, Jefferson Co. | 65.3 |
| edwa_MtLi_1 | RIH2768 | *edwardsii* |  | USA, CO, Jefferson Co. | 108.2 |
| egeg_108m_1 | RIH2143 | *egleis* | *egleis* | USA, CA, Alpine Co. | 55.7 |
| egeg_108E_1 | RIH2142-B | *egleis* | *egleis* | USA, CA, Mono Co. | 114.2 |
| egow_Juni_1 | CRT303 | *egleis* | *oweni* | USA, CA, Plumas Co. | 95.8 |
| egow_Juni_2 | CRT277 | *egleis* | *oweni* | USA, CA, Plumas Co. | 63.5 |
| noap_Brid_1 | RIH2212 | *nokomis* | *apacheana* | USA, CA, Mono Co. | 70.5 |
| noap_Brid_2 | RIH2112 | *nokomis* | *apacheana* | USA, CA, Mono Co. | 14.7 |
| noca_FoRd_1 | CRT465 | *nokomis* | *carsonensis* | USA, NV, Douglas Co. | 66.7 |
| noca_FoRd_2 | CRT466 | *nokomis* | *carsonensis* | USA, NV, Douglas Co. | 24.8 |
| hedo_BlyK_1 | RIH2971 | *hesperis* | *dodgei* | USA, OR, Klamath Co. | 89.6 |
| hedo_BlyK_2 | RIH2969 | *hesperis* | *dodgei* | USA, OR, Klamath Co. | 48.1 |
| hedo_FrNF_1 | CRT383 | *hesperis* | *dodgei* | USA, OR, Lake Co. | 31.0 |
| hedo_FrNF_2 | CRT385 | *hesperis* | *dodgei* | USA, OR, Lake Co. | 64.1 |
| hyhy_Bowm_1 | RIH2663 | *hydaspe* | *hydaspe* | USA, CA, Nevada Co. | 59.0 |
| hyhy_Bowm_2 | RIH2664 | *hydaspe* | *hydaspe* | USA, CA, Nevada Co. | 86.3 |
| hyvi_Mill_1 | RIH2149 | *hydaspe* | *viridicornis* | USA, CA, Tuolumne Co. | 43.5 |
| hyvi_Mill_2 | RIH2151 | *hydaspe* | *viridicornis* | USA, CA, Tuolumne Co. | 30.8 |
| idid_FrIn_1 | WS13-04 | *idalia* | *idalia* | USA, PA, Lebanon Co. | 19.2 |
| idid_FrIn_2 | WS13-03 | *idalia* | *idalia* | USA, PA, Lebanon Co. | 43.1 |
| idid_FrIn_3 | WS13-02 | *idalia* | *idalia* | USA, PA, Lebanon Co. | 169.4 |
| idid_FrIn_4 | WS13-05 | *idalia* | *idalia* | USA, PA, Lebanon Co. | 59.3 |
| momo_Iceh_1 | RIH2580 | *mormonia* | *mormonia* | USA, CA, El Dorado Co. | 104.1 |
| momo_Sher_1 | CRT234 | *mormonia* | *mormonia* | USA, CA, Tulare Co. | 96.1 |
| moob_InNF_1 | CRT463 | *mormonia* | *obsidiana* | USA, CA, Mono Co. | 88.9 |
| moob_InNF_2 | CRT464 | *mormonia* | *obsidiana* | USA, CA, Mono Co. | 85.3 |
| zemo_InLk_1 | RIH2063 | *zerene* | *monticola* | USA, CA, Nevada Co. | 30.8 |
| zemo_Stra_1 | RIH2254 | *zerene* | *monticola* | USA, CA, Tuolumne Co. | 79.2 |
| zemo_Stra_2 | RIH2252 | *zerene* | *monticola* | USA, CA, Tuolumne Co. | 134.3 |
| zemo_Stra_3 | RIH2253 | *zerene* | *monticola* | USA, CA, Tuolumne Co. | 55.2 |
| Arze_Chin_1 | RIH3741 | *zenobia* |  | China | 6.8 |
| Fcor_Russ_1 | RIH3743 | *coredippe* |  | Russia | 13.7 |
| Fjai_Indi_1 | RIH3738 | *jainadeva* |  | India | 2.9 |
| Fjai_Indi_2 | RIH3739 | *jainadeva* |  | India | 7.4 |
| Fner_Chin_1 | RIH3740 | *nerippe* |  | China | 8.3 |
| clar_Chin_1 | RIH3735 | *clara* |  | China | 21.0 |
| clar_Chin_2 | RIH3736 | *clara* |  | China | 12.5 |
| clar_Chin_3 | RIH3737 | *clara* |  | China | 16.3 |
