# Supplementary material for: Phylogenomic Analyses Clarify True Species within the Butterfly Genus Speyeria despite Evidence of a Recent Adaptive Radiation
Source: Insects. 2019 Jul 17;10(7):209. doi: 10.3390/insects10070209 (PMC6681192; doi:10.3390/insects10070209)
Supplement: Supplementary file 1 [file insects-10-00209-s001.zip › insects533876-suppl/Table S2.docx]

**Table S2.** Information about each locus analyzed, and the evolutionary model selected.

| **Locus Name** | **Minimum Sequence Length (BP)/Individual** | **Evolutionary Model** |
| --- | --- | --- |
| R000958 | 177 | HKY+G |
| R000975 | 99 | HKY+G |
| R001081 | 221 | HKY+G |
| R001084 | 173 | HKY+G |
| R001097 | 160 | F81 |
| R001106 | 268 | HKY |
| R001113 | 201 | HKY+I+G |
| R001129 | 270 | SYM+I+G |
| R001192 | 240 | HKY+I |
| R001199 | 227 | HKY+I+G |
| R001218 | 221 | GTR+G |
| R001221 | 207 | HKY+I+G |
| R001222 | 203 | HKY+I |
| R001237 | 199 | GTR |
| R001247 | 253 | K80+I+G |
| R001257 | 99 (0 for 1 outgroup individual) | GTR+I+G |
| R001264 | 235 | HKY+I |
| R001278 | 131 | GTR+I+G |
| R001291 | 229 | HKY+G |
| R001295 | 170 | HKY+G |
| R001297 | 239 | HKY |
| R001307 | 194 | GTR+G |
| R001373 | 222 | GTR+G |
| R001386 | 241 | SYM+I |
| R001390 | 223 | HKY+G |
| R001460 | 173 (0 for 1 *hydaspe* individual) | HKY+G |
| R001475 | 195 | HKY+I |
| R001487 | 236 | GTR+I+G |
| R001501 | 173 | GTR |
| R001591 | 198 | HKY+G |
| R001620 | 159 | HKY+G |
| R001697 | 211 | HKY+G |
| R001768 | 272 | HKY+G |
| R001817 | 162 | HKY+G |
| R001838 | 263 | GTR+I |
| **Locus Name** | **Minimum Sequence Length (BP)/Individual** | **Evolutionary Model** |
| R001844 | 250 | SYM+G |
| R001916 | 187 | GTR+I+G |
| R001937 | 148 | GTR+I+G |
| R001944 | 233 | GTR+G |
| R001966 | 202 | HKY+G |
| R001976 | 217 | GTR+G |
| R001993 | 232 | HKY+G |
| R002003 | 256 | SYM+I |
| R002040 | 187 | HKY+I+G |
| R002061 | 136 | HKY+G |
| R002091 | 170 | HKY+G |
| R002232 | 294 | GTR+G |
| R002382 | 135 | HKY+I |
